# Esports in-game consumption across generations: Integrating motivated reasoning theory and the theory of planned behavior

**DOI:** 10.1371/journal.pone.0348355

**Published:** 2026-05-04

**Authors:** Kun Chang, Jun-Phil Uhm, Hyun-Woo Lee

**Affiliations:** 1 Department of Sport and Entertainment Management, University of South Carolina, Columbia, South Carolina, United States of America; 2 Department of Kinesiology, Inha University, Incheon, Republic of Korea; 3 Department of Kinesiology & Sport Management, Texas A&M University, College Station, Texas, United States of America; Xuzhou University of Technology, CHINA

## Abstract

The revenue from virtual non-functional products has become a significant revenue source for the video game industry. Despite growing interest, understanding the decision-making processes behind virtual non-functional products consumption in free-to-play games remains limited. This study contributes to existing literature by testing a multi-relational model (motivation-cognition-intention-behavior). We aim to examine how gamers’ self-presentation motivation is associated with their attitude, subjective norm, and perceived behavioral control, and how these factors relate to their purchase behavior. Data were collected via an online survey distributed on Reddit to esports gamers with prior purchase experience (*N* = 239). All constructs were measured using established multi-item Likert scales. Structural equation modeling and multi-group analysis were conducted using Mplus 8.8. Results showed that gamers’ self-presentation motivation in the game is positively associated with gamers’ attitude (*β* = .246, *p* < .001) and subjective norm (*β* = .169, *p* < .05). Attitude (*β* = .398, *p* < .001), subjective norm (*β* = .155, *p* < .05), and perceived behavioral control (*β* = .224, *p* < .001) were positively associated with purchase intention, which in turn predicted purchase behavior (*β* = .600, *p* < .001). Further, we identified generational differences on the path of motivation-cognition-intention-behavior. The current study moves beyond a single-theory explanation by proposing a more holistic perspective of how gamers’ innate self-presentation motivations are associated with virtual non-functional products consumption. The findings revealed novel insights into generational differences in the decision-making processes, guiding tailored marketing strategies to boost virtual non-functional products consumption across segments.

## Introduction

Virtual non-functional products (NFPs) refer to purely ornamental items in online video games, such as avatar skins or cosmetics, changing only the visual appearance of a gamer’s avatar or character and associated artifacts or possessions [1]. These products offering no gameplay advantages have emerged as a major income stream for the rapidly developing online video gaming industry [2,3]. The growing market for NFPs, consumed primarily by Gen Z and Millennials, presents significant theoretical and practical implications for consumer behavior and product management within virtual marketplaces. Although this phenomenon is gaining scholarly interest, empirical understanding of how motivation shapes decision-making processes and how these processes differ across generations in the context of NFPs remains limited.

Scholars have conceptualized and investigated various factors influencing in-game products consumption, including value-based factors such as entertainment values and social values [4–6], behavioral factors such as brand engagement [7], and motivational factors such as coolness and joy [8]. A growing body of research suggests that players purchase NFPs are associated not only with entertainment but also with their digital identity expression, as in-game avatars serve as a key medium for self-expression in virtual spaces [2,5,9,10]. Specifically, the study emphasized self-presentation as a core motivation, particularly in multiplayer game environments where social interaction is prominent [1].

While existing studies provide valuable insights into NFPs consumption, they often rely on single-theory explanations rather than comprehensive models that account for various influencing factors that are often interconnect. Specifically, studies based on motivational theories often neglect how motives may relate to cognitive factors l purchase behavior; conversely, cognitive-based models tend to overlook the intrinsic motivations that drive consumer decisions [1,5,9]. Given the complexity of virtual social environments and established evidence linking motivation and cognition, a more holistic, multi-relational approach is necessary to provide a more comprehensive view of esports consumers’ NFP purchase behavior.

The current study developed a model synthesizing the theory of planned behavior (TPB) [11] with motivated reasoning theory (MRT) [12]. As one of the most effective cognitive-based frameworks established to explain behavior, the TPB has been applied to explain how people’s attitude, SN, and PBC are associated with their intentions and behaviors in various settings, including the purchase of in-game products [13]. As MRT postulates that people’s motivations are closely associated with how they think, perceive, and feel, we examined how virtual self-presentation motivation (VSPM) relates to purchase through attitude, subjective norm (SN), perceived behavioral control (PBC), and intention based on the TPB. Moreover, although Generation Z (Gen Z) and Millennials are the primary demographic groups purchasing in-game skins, their shared market relevance does not imply that the underlying decision-making process is the same [14,15]. For example, compared to Millennials, Gen Zs are reported to have a stronger attachment to their game characters and are relatively more autonomous in purchasing in-game accessories for their avatars [16–18]. The theoretical boundary conditions of the hypothesized model are assessed by testing the generational differences.

The purpose of this study was to, therefore, examine how gamers’ self-presentation motivation in the game is associated with their attitude, SN, and PBC, and how these factors relate to purchase intention and consumption of NFPs. We recruited esports gamers who previously purchased in-game products from various social media platforms and tested how the relations may differ across Gen Zs and Millennials. We expect that our findings will offer valuable insights for researchers and practitioners focused on consumer behavior and management of virtual products in online video game marketplaces. The motivation-cognition-intention-behavior model of NFPs consumption is empirically supported. Highlighting generational differences in the pattern of relationships associated with purchase offers practical contributions, enabling customized marketing strategies to satisfy the distinct needs of each generation. In addition, modeling purchase itself has empirical value as scholars rarely include purchase behavior in their empirical studies, despite the extensive utilization of the TPB and theoretical and practical values of assessing actual behavior [19].

## Theoretical background

### The theory of planned behavior

The TPB is an influential theory in consumer behavior that explains the association between an individual’s beliefs and their behaviors [11]. This theory ascribes an individual’s consumption, behavioral intentions, and subsequent behavior as determined by attitude, SN, and PBC. TPB has been widely applied in various research topics related to fan behaviors, including both physical goods or service consumption and digital consumption [10,20]. We adopted TPB as the theoretical basis for our study, as it can help identify the underpinnings of intent to consume (i.e., attitude, SN, and PBC) and lays the foundation for explaining purchase behavior. In line with the TPB, attitude and SN are specified as predictors of behavioral intention, which in turn serves as the most proximal determinant of behavior [11]. These constructs are generally modeled as influencing behavior indirectly through intention. In contrast, PBC may exert both indirect effects (via intention) and a direct effect on behavior when it reflects actual control [11]. Accordingly, we include a direct path from PBC to purchase, while modeling attitude and SN as indirect predictors of behavior through intention. Although TPB has provided vast theoretical and practical insights for understanding purchase behavior as a cognitive-based theoretical framework, it may be limited in explaining how an individual’s planned behaviors are evaluated and determined by their motives [10,21]. Incorporating motivational factors into a cognitive-based perspective (i.e., TPB) is instructive for a more comprehensive understanding of NFPs consumption [22].

### The motivated reasoning theory

The MRT provides an important theoretical background on bridging the relationship between an individual’s motivations and their cognitive responses [12]. According to this theory, individuals in pursuit of desired outcomes—an individual’s desire to arrive at positively self-serving conclusions—might motivate them to perceive information in a manner most beneficial to them [12]. This process involves searching for and selecting only a subset of memories and evidence that construct the desired belief. In this sense, an individual’s motives are likely to be associated with their attitudes, beliefs, and perceptions. The MRT has been employed across various social and psychological settings, demonstrating its ability in explaining how motivation is associated with cognitive status, and how such association relates to subsequent behaviors. However, similar to TPB, which emphasizes cognition-behavior, the MRT focuses on the motivation-cognition process that offers only partial insight, which likewise only holds a segmented perspective that is deemed insufficient in holistically understanding consumer behavior [22].

### The integration of TPB and MRT

From the perspective of motivated reasoning, individuals’ underlying motives could potentially bias the way they attend to, interpret, and evaluate information relevant to a focal behavior [12]. In the context of NFPs purchasing, gamers with stronger virtual self-presentation motivation may selectively attend to information that frames NFPs as identity-enhancing or socially valued, while discounting information that emphasizes their non-functional nature. Through this motivated cognitive process, such motivations become systematically associated with more favorable attitudes toward NFPs purchase (attitude), heightened perceptions of social approval (SN), and stronger perceived alignment between the behavior and desired self-presentation outcomes (PBC). In this way, MRT provides a theoretical foundation for understanding how motivational states correspond to the attitudinal and normative components specified by the TPB. While the TPB explains how attitude, SN, and PBC are associated with intention and behavior, it does not explicitly address how these cognitive evaluations are formed. MRT complements this by explaining how underlying motivations are associated with the formation of these cognitive evaluations. In this sense, MRT provides a mechanism linking motivation to the cognitive components specified in the TPB.

**Fig 1:**
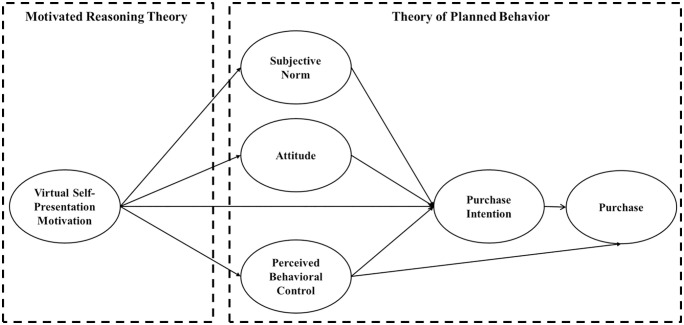
Theoretical Framework. *Note.* Multi-group (Gen Zs vs. Millennials) was included in this model to compare the process of motivation-cognition-intention behavior.

Building on the synthesis of TPB and MRT, the current study offers a multi-relational perspective (i.e., motivation-cognition-intention-behavior) as opposed to assessing a unicausal model of NFPs consumption. The current study applies the TPB framework while incorporating insights from MRT to explain how motivational factors are associated with cognitive evaluations in the context of NFPs consumption. Specifically, we hypothesize and examine the relationship between VSPM and their cognitive responses (i.e., attitude, SN, and PBC) (see Fig 1).

In addition, while TPB has been applied extensively in consumer behavior studies in many contexts, the majority of previous studies have been restricted to the relationship between consumers’ attitudes, SN, PBC, and behavioral intentions, without any consideration of their actual execution of those behaviors. Thus, the current study tests purchase as the subsequent variable of purchase intention. To that end, we found that the TPB model was augmented by integrating both motivational factors (i.e., VSPM) and purchase within the context of NFPs consumption. It, thereby, provides a more comprehensive explanation of the process of consumption behavior formation. In the following section, we reviewed the relationships among the constructs and developed hypotheses.

## Hypothesis development

### Virtual self-presentation motivation and intention to purchase NFPs

VSPM refers to the desire to convey a favorable image to others in the virtual environment [1]. To manage how they are perceived by others, its objective is to display an affirmative image to others and make the desired impression on them by adjusting their personal presentation in a certain environment [23]. Individuals strive to express themselves in a way reflecting positively on them, and they use various resources to give others the impression they desire to convey [1,24].

Previous research has emphasized that although NFPs do not directly add value to gamers’ game performance, they possess expressive value and purpose, which can enhance gaming experiences [25]. Accordingly, VSPM is considered as a particularly significant factor in predicting NFPs consumption in various online gaming environments [5,26]. Indeed, game characters are central for gamers to manifest and manage their personal images, substantially intensifying their means of information presentation and changing their expression of their original identity. Purchasing virtual enhancements, such as clothing and accouterments, to modify their in-game characters and weapons allows gamers to better express themselves and interact with others in the virtual environment [2]. Researchers found that users’ self-expression in social networking service games is positively correlated with their purchase of digital goods [1]. Accordingly, we posit a positive association between VSPM and the intention to purchase NFPs in the video game context:

H1: VSPM is positively associated with the intention to buy NFPs.

### The Mediating Role of Attitude, Subjective Norms, and Perceived Behavioral Control

In accordance with definitions under TPB and existing literature, our study operationally defines attitude, SN, and PBC as follows [11]. Attitude represents an individual’s perspectives (positive or negative) regarding the in-game purchase of in-game cosmetic products, and their personal views of such acts. SN refers to the consensual acceptance of purchasing NFPs by a reference group (e.g., peers and people of importance to the person). Finally, PBC describes an individual’s perceptions of the availability or lack of resources or opportunities required to prompt in-game purchasing of cosmetic products. According to TPB, attitude, SN, and perceived PBC are key determinants of behavioral intention [11]. For example, a consumer’s pre-existing positive or negative dispositions towards purchasing products are strongly linked to their purchasing intent. Furthermore, gamers’ beliefs about whether the majority of people approve or disapprove of purchasing NFPs, as well as perceptions of the difficulty or ease of acquiring those NFPs, are also linked to purchase intention [13]. PBC is evaluated by a set of control beliefs about the presence of certain factors which may promote or hinder the performance of a particular behavior and is thus strongly associated with an individual’s intentions [11]. That is, the more resources and fewer obstacles an individual perceives in their purchasing process, the greater their PBC and the stronger their intention to perform specific behaviors. Together, individuals with more favorable attitudes toward a behavior, stronger perceived social approval, and greater perceived control are more likely to form stronger intentions to perform that behavior. Accordingly, we propose that: 1) attitude is positively associated with NFPs intention purchase, 2) SN is positively associated with NFPs intention purchase, and 3) PBC is positively associated with NFPs intention purchase.

The MRT posits that motivation is associated with reasoning through a reliance on a biased set of cognitive processes, which are strategies for accessing, constructing, and evaluating beliefs [12]. The theory proposes that motivation can enhance the use of beliefs and evaluations which are considered to be most appropriate and the most likely to yield desired outcomes. This theory also describes that people are more likely to reach conclusions that reflect their beliefs, but their ability to do so is constrained by their ability to construct seemingly reasonable justifications for these conclusions. Based on the cognitive mechanisms described by the MRT, motivated reasoning offers a mechanism through which self-presentation motivation becomes associated with the attitudinal, normative, and control beliefs specified in the TPB. Specifically, through this motivated reasoning process of selectively attending to and interpreting information, individuals may evaluate NFPs more favorably (attitude), perceive greater social approval from others (SN), and feel greater ease or ability to engage in purchasing (PBC). Thus, we posit that people’s motivation to present themselves in an in-game environment can influence: (1) their attitude toward purchasing NFPs, (2) a belief determined by significant others, and (3) the perception that an individual’s behavior can determine whether they can attain the desired outcome [12]. Combining these relationships, we propose that VSPM is indirectly associated with purchase intention through cognitive processes and hypothesize that:

H2: Attitude, SN, and PBC will mediate the relationship between VSPM and intention to buy NFPs.

### Direct association between intention and behavior

According to the TPB, the stronger an individual’s intention to perform a particular behavior, the greater the likelihood that the person will perform that behavior [11]. A study found that gamers’ consumption intention reflects their actual consumption of playing [27]. Thus, the current study operationally considered gamers’ purchase rate as the frequency of purchasing in-game skins. Nevertheless, most studies based on the TPB did not examine actual behavior due to inherent difficulties in measuring it [19]. A study conducted a meta-analysis on in-game purchase studies and concluded that most of the research studies rarely examined purchase behavior [28]. Furthermore, studies examining whether behavioral intentions predict actual behavior reported different results in different contexts. For example, some studies found a marginal association between intention and consumption behavior, whereas others documented a stronger association between purchase intention and purchasing of NFPs [13,21]. To resolve this ambiguity, we investigated whether intention is linked to purchase behavior based on the conceptual model of the TPB in the video game context, and established the following hypothesis:

H3: Purchase intention is positively associated with purchase of NFPs.

### Direct association between perceived behavioral control and purchase

According to TPB, an individual’s beliefs about their abilities to perform a specific behavior of interest are associated with whether they actually engage in that behavior [11]. A key prerequisite for PBC, in most instances described by the TPB, is an individual’s confidence in their ability to perform specific behavior. For example, when people are relatively confident about the outcome of their own behavior, they will be less likely to acquire and interpret information based on the conduct or opinion of others (e.g., social norms) as the primary source of information guiding their behaviors [11]. In a similar vein, regarding purchasing behavior in a virtual setting, if an individual is confident in their ability to engage in online purchasing, they can feel positively about their behavioral control when making purchases in a virtual setting [29]. Thus, we established the following hypothesis:

H4: PBC is positively associated the purchase of NFPs.

### The generational differences: Gen Zs vs. Millennials

Given that Gen Zs and Millennials are the main consumer base in the online gaming industry, the theoretical and practical significance of understanding the consumption patterns of this population has been underscored by scholars and practitioners [30]. Drawing from generational theory [14], each generation has its own values, beliefs, motivations, experiences, and expectations that define the identity of the generation and are associated with their consumption behaviors. These cohort-based differences are particularly relevant in digital environments, where identity expression and social interaction are central, and may influence how motivation relates to cognitive evaluations and subsequent behavior [15,31]. According to the research, Gen Zs are those born between 1997–2012, and Millennials are those born between 1981–1996 [32]. While both generations are heavily involved with modern technological experiences, Gen Zs are reckoned as the true natives of the digital era as they have grown up with advanced technology since birth [33].

In comparison to Millennials, Gen Zs have themselves grown up in a more mature, diverse, competitive, and immersive video gaming environment, and thus, exhibited different psychological and behavioral patterns that are potentially associated with their in-game consumption behaviors [34]. According to the study, members of Gen Zs not only enjoy and try to master games but also have stronger self-identities with the game characters and use games as spaces to interact with others more heavily [17]. The report shows that, in general, Gen Zs are more likely to purchase video game currencies and clothing or accessories for their avatar than other populations [18]. In this sense, Gen Zs have relatively higher autonomy in developing their game characters and a stronger motivation to self-present through these game characters [35].

On the other hand, TPB suggests that people’s cognitive processes (e.g., beliefs) may vary across demographic factors such as age, and that these factors are indirectly associated with behavior through their relationships with cognitive processes [36]. Following this logic, there may be differences in the relationship between cognitive pathways (i.e., attitudes, SN, and PBC) and their purchase intention of NFPs across generations. Previous studies have also examined and suggested that generational-based differences are associated with variation in people’s motivated reasoning process overall [37]. Thus, generational cohort may influence how motivation is associated with cognitive evaluations and how these evaluations relate to intention and behavior in the context of NFPs consumption. Accordingly, we hypothesize that:

H5: There will be group differences in motivation-cognition-intention process behind NFPs consumption between Gen Zs and Millennials.

## Method

### Participants

Data were collected via an online survey facilitated by Qualtrics and distributed on Reddit.com from March 22 to March 31, 2022, and accessed on April 1, 2022. The sampling frame consisted of esports gamers who had prior experience purchasing NFPs. Informed consent was obtained digitally from all participants as a condition of entry into the study. The procedure presented a detailed information sheet outlining the study’s purpose, procedures, and participant rights, after which individuals provided explicit consent to proceed. This study protocol, including the consent procedure, was reviewed and granted an exemption by the institutions’ Institutional Review Board. Specifically, participants were recruited through postings on the subreddits of major video games characterized by high levels of interaction among members, including Counter-Strike: Global Offensive/ CS: GO, DOTA, League of Legends/ LOL, and Fortnite. Each subreddit had about 5,000–1,000,000 members at the time of data collection. Participation was voluntary, and respondents accessed the survey through a link provided in these posts. The survey began with four screening questions to ensure that prospective participants were gamers and had experience purchasing NFPs. We also utilized the reCAPTCHA fraud and bot detection system to protect against collecting false data, eliminating invalid responses from the collected data. Of the 467 initial respondents, 239 were deemed viable participants: 204 males (85.4%) and 35 (14.6%) females. There were 138 participants who were Gen Zs and 101 participants were Millennial, with an average age of 25 (*SD* = 5.407). Among the participants, 146 participants (61.1%) reported playing first-person shooter games (e.g., CS: GO), and 93 (38.9%) reported playing multiplayer online battle arena games (e.g., Dota and League of Legends). Most participants were white or Caucasian (*n* = 159, 66.5%), followed by Black or African American (*n* = 38, 15.9%) and Asian (*n* = 23, 9.6%). Most had a bachelor’s degree or above (*n* = 166, 69.4%). This study was approved by the Institutional Review Board at Texas A&M University (IRB2022-0094M).

### Measures

The measurement scales were adapted from prior validated studies as they capture the same underlying constructs examined in this research. Minor wording adjustments were made to reflect the context of NFPs consumption while preserving the original conceptual meaning of each construct. We adopted four items from [5] to measure participants’ motivation to present themselves in the virtual gaming environment. Attitudes and SN were measured using three items each, adopted from the study [20]. Kim and Karpova’s [38] scale with three items were employed to measure PBC. Finally, three items measuring intention to purchase NFPs and two items measuring self-reported purchase of the NFPs were adopted from existing studies [39,40]. All measures were gauged using a seven-point Likert scale ranging from 1 (strongly disagree) to 7 (strongly agree), and the wording of the items is presented in the Appendix.

The assumption of normality was assessed using skewness and kurtosis before testing the hypotheses. The skewness ranged from −.097 to −1.576, and kurtosis ranged from −1.171 to 5.544. Each of these values fell within the recommended ranges from −2 to +2 and from −7 to +7 [41] confirming the normal distribution of the variables. The results of the confirmatory factor analysis provide support for the validity and reliability of the adapted measures in this context.

### Common method bias

To address the potential for common method bias in the survey design, we performed Harman’s one-factor test, in which all the measures in the study are subjected to exploratory principal component factor analysis. Evidence of common method bias is indicated when any single factor emerging from the analysis accounts for more than 50% of the variance [42]. The result showed that a single factor accounted for 26.53% of the variance in the data. As no single factor accounted for more than 50% of the covariance in the independent and criterion variables, we concluded that the data did not indicate common method bias. Procedural steps were taken to minimize common method bias, including ensuring respondent anonymity and using established measurement scales to reduce respondents’ ability to infer relationships among variables.

### Data analysis

We conducted a confirmatory factor analysis (CFA) to assess the measurement model. Absolute fit indices of standardized root mean square residual (SRMR) and root mean square error of approximation (RMSEA), and incremental fit indices of the comparative fit index (CFI) and Tucker–Lewis index (TLI) were computed to assess the fit of the model [43]. Convergent and discriminant validity was tested by computing composite reliability (CR) coefficients and average variance extracted (AVE) values, then comparing the square root of AVE against the multiple correlations [44]. Following this, structural equation modeling (SEM) was employed to test the hypothesized relationships among the constructs, including both direct and indirect effects. Multi-group analysis was conducted to examine whether the structural relationships differed across Generation Z and Millennials. We conducted 10,000 iterations of bootstrapping to test indirect relationships and also measured bias-corrected percentile intervals [45]. We used SPSS Version 28.0 (IBM Corp., Armonk, NY) for descriptive statistics and used Mplus 8.8 for data analysis at an alpha level of.05 [46]. Mplus was selected due to its flexibility in estimating structural equation models with bootstrapping and multi-group analysis.

## Results

### Structural model assessment

The measurement model indicated an acceptable fit of the data (*χ*^2^ = 201.957, *df* = 120, TLI = .958, CFI = .967, SRMR = .056, RMSEA = .053). All factor loadings exceeded the minimum recommended threshold of.50, indicating acceptable indicator reliability [41]. The psychometric properties and factor correlation coefficients for each latent variable are reported in Table 1 and Table 2. Factor loadings, AVE, and CR coefficient values supported evidence of convergent validity.

**Table 1:** Psychometric Properties of the Measurement Model.

Constructs and Items	*λ*	*SE*	*ρ*	AVE
Virtual Self-Presentation Motivation			.853	.594
VSPM 1	.722	.037		
VSPM 2	.886	.026		
VSPM 3	.763	.033		
VSPM 4	.697	.040		
Attitude			.858	.669
ATT1	.772	.035		
ATT2	.826	.029		
ATT3	.854	.027		
Subjective Norm			.802	.579
SN1	.598	.030		
SN2	.852	.027		
SN3	.809	.027		
Perceived Behavioral Control			.763	.520
PBC1	.624	.052		
PBC2	.774	.050		
PBC3	.757	.049		
Intention to Purchase			.956	.878
INT1	.959	.009		
INT2	.957	.009		
INT3	.894	.015		
Purchase			.888	.799
P1	.867	.029		
P2	.919	.027		

*Note*. *λ* = factor loadings; *SE* = standard error; *ρ* = composite reliability; AVE = average variance extracted; all *p-value* < .001.

**Table 2:** Factor Correlations.

	1	2	3	4	5	6
1. VSPM	(.771)					
2. ATT	.247***	(.818)				
3. SN	.169*	.347***	(.761)			
4. PBC	−.097	.006	−.006	(.721)		
5. INT	.208***	.472***	.309***	.209***	(.937)	
6. Purchase	.277***	.538***	.203***	.014	.596***	(.894)

*Note*. Number in parentheses in diagonal are square roots of average extracted variance for each construct. VSPM = virtual self-presentation motivation; ATT = attitude; SN = subjective norm; PBC = perceived behavioral control; INT = intention to purchase NFPs *p < .05, ***p < .001.

**Fig 2:**
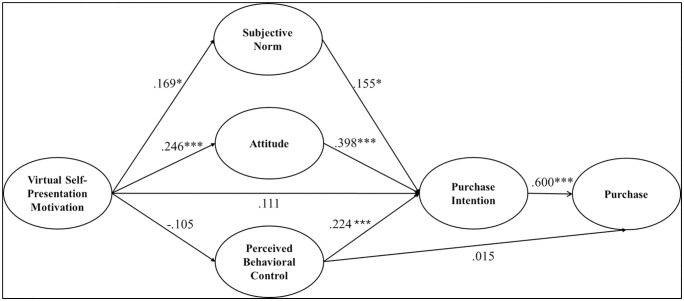
Path Diagram of the Structural Equation Model. *Note.* Multi-group (Gen Zs vs. Millennials) was included in this model to compare the process of motivation-cognition-intention behavior. *p < .05, ***p < .001.

The structural model indicated an acceptable fit of the data (χ^2^ = 221.326, *df* = 122, TLI = .949, CFI = .960, SRMR = .064, RMSEA = .058). All direct and indirect paths are reported in Table 3. The results showed that the direct paths from VSPM to purchase intention and from PBC to purchase were nonsignificant, rejecting Hypothesis 1 and Hypothesis 4. However, intention to purchase had a significant and direct association with purchase, supporting Hypothesis 3. In addition, the indirect path through attitude and purchase intention between VSPM and purchase was significant (*b* = .062, *SE* = .021, *p* = .003). However, the indirect association through SN and PBC between VSPM and intention to purchase was nonsignificant. Fig 2 provides a visual representation of the structural equation model, depicting the estimated relationships among the study variables.

**Table 3:** Results of Direct and Indirect Paths.

	Standardized	BC Interval
*Direct Effects*		
VSPM→ATT	.246***	[.114,.392]
VSPM→SN	.169*	[.024,.314]
VSPM→PBC	−.105	[-.251,.055]
VSPM→INT	.111	[-.024,.229]
ATT → INT	.398***	[.267.529]
SN → INT	.155*	[.022.289]
PBC → INT	.224***	[.095.348]
INT → P	.600***	[.489.691]
PBC → P	.015	[-.234.030]
*Indirect Effect*		
VSPM→ATT → INT	.106***	[.035.166]
VSPM→SN → INT	.026	[-.006.058]
VSPM→PBC → INT	−.022	[-.058.015]

*Note*. VSPM = virtual self-presentation motivation; ATT = attitude; SN = subjective norm; PBC = perceived behavioral control; INT = intention to purchase NFPs; P = purchase; Reported BC Intervals are the bias corrected 95% confidence interval of standardized estimates resulting from bootstrap analysis; *p < .05, **p < .01, ***p < .001.

### Multi-group analysis

Multi-group analysis was performed to test whether there is a difference in the path coefficients of the research model determined by generations. We tested for configural invariance, metric invariance, and scalar invariance before conducting multigroup analysis. As shown in Table 4, the configural model indicated an acceptable fit of the data, indicating that configural invariance was supported. Chi-square difference tests showed the difference between the fit of configural and metric models was nonsignificant (Δ*χ*^2^ ＝ 18.713, Δ*df* ＝ 12, *p* = .096), indicating that metric invariance was supported. The difference between the fit of metric and scalar models was also nonsignificant (Δ*χ*^2^ ＝ 14.571, Δ*df* ＝ 12, *p* = .266), indicating that scalar invariance was also supported.

**Table 4:** Results of Multigroup Analysis.

Path	Standardized (*β*)		Δ	*df*	*p*
	Gen Z	Millennial			
VSPM→ATT	.292**	.185	4.351*	1	.037
VSPM→SN	.080	.312**	6.318*	1	.012
VSPM→PBC	−.129	−.088	4.219*	1	.040
VSPM→INT	.160	−.011	5.933*	1	.015
ATT → INT	.493***	.347***	5.236*	1	.022
SN → INT	.106	.273**	5.835*	1	.016
PBC → INT	.168	.300**	5.209*	1	.022
INT → P	.663***	.590***	4.755*	1	.029
PBC → P	−.055	−.196	5.412*	1	.020

*Note*. VSPM = virtual self-presentation motivation; ATT = attitude; SN = subjective norm; PBC = perceived behavioral control; INT = intention to purchase NFPs; P = purchase; Reported BC Intervals are the bias corrected 95% confidence interval of standardized estimates resulting from bootstrap analysis; *p < .05, **p < .01, ***p < .001.

As configural invariance, metric invariance, and scalar invariance were supported, we examined the existence of a difference in the path coefficients determined by generations and tested group differences by constraining each path. The results of the Chi-square difference tests were reported in Table 4, showing all paths were significantly different between the Gen Z group and the Millennial group, supporting Hypothesis 5. The multi-group analysis revealed distinct patterns across the two groups. Specifically, the indirect association between VSPM and purchase through attitude and purchase intention was only significant for the Gen Z group (*b* = .095, *SE* = .037, *p* = .010); whereas this indirect association with purchase through SN and purchase intention was only significant for the Millennial group (*b* = .063, *SE* = .031, *p* = .043). As shown in Table 4, for Generation Z, VSPM was more strongly associated with attitude, and attitude shows a stronger relationship with purchase intention. For Millennials, VSPM was more strongly associated with SN, and SN has a stronger relationship with purchase intention. In addition, PBC has a stronger effect on intention among Millennials, whereas the relationship between intention and purchase is stronger among Generation Z.

## Discussion

The current study sheds light on the relationship among VSPM, attitudes, SN, PBC, generation difference, purchase intention, and purchase. According to our findings, attitude was a significant mediator between VSPM and intention to purchase, while SN and PBC were not. These findings are consistent with the theoretical integration of MRT and TPB, suggesting that motivated reasoning is highly associated with gamers’ favorable attitude and perceived norm that are central to behavioral intention. However, these relationships vary based on generations. Specifically, we found that Generation Z and Millennials demonstrated different patterns regarding the mediation role of attitude and SN on the relationship between VSPM and purchase intention. Finally, we found that purchase intention is favorably linked to purchase, whereas the direct relationships between VSPM and purchase intention, and between PBC and purchase were nonsignificant. A detailed discussion of the findings and contributions was presented in the following sections.

Specifically, our results showed that the direct association between VSPM and intention was statistically nonsignificant, rejecting Hypothesis 1. That is, gamers’ motivation to present themselves in games is not directly associated with their intention to purchase NFPs when other variables are considered. This contradicts the findings of most prior studies on in-game consumption in the general video gaming field, which suggest that gamers’ self-expression in online communities is positively associated with their intention to purchase virtual goods [1]. One possible explanation is that motivation alone may not be sufficient to predict behavioral intention in this context. Rather, its effect appears to operate through cognitive evaluations, particularly attitude, which serves as a more proximal predictor of intention. The addition of cognitive factors to our model therefore provides a more rigorous explanation of how VSPM is associated with purchase intention in the context of NFPs consumption.

VSPM among gamers is highlighted as an important factor of in-game cosmetic item purchasing. Interpreting these results from the perspective of MRT, VSPM is strongly and positively associated with gamers’ cognitive processes that correspond to more favorable attitudes toward purchasing NFPs as a means of self-presentation in the game. Thus, gamers’ positive attitude is linked with a higher likelihood of purchasing NFPs, which is consistent with TPB reasoning that favorable evaluations of products tied to self-presentation relate to purchase behavior. In a virtual-based social environment, the appearance of their avatar and their social relationships are essential to gamers’ gaining attention, gaining popularity, and forming social connections. Therefore, it is reasonable that gamers who are more motivated to express their preferred self-images in games will consider purchasing NFPs as a critical factor of their optimal gaming experience. Previous studies in various disciplines also supported the same notion illustrating the positive relationship between an individual’s motivation, cognitive responses, and purchase intention [1,28,47].

Interestingly, this significant mediating role of attitude in the association between virtual self-presentation motivation and purchase intention was only found in the Gen Z group, but not in the Millennial group. This indicates that Gen Zs may place greater emphasis on their own thoughts and feelings about purchasing NFPs, whereas others’ opinions are less strongly associated with their purchase intention. What is more interesting is that, while the indirect association of SN between VSPM and purchase intention was marginal and statistically insignificant in general, we did find an age-based difference in this relationship. On the other hand, the positive mediation role of SN on the relationship between VSPM and purchase intention was only significant in the Millennial group. That is to say, unlike Gen Zs, Millennials may be more influenced by how others think about purchasing NFPs. These findings echo the theoretical proposal of the TPB, suggesting that an individual’s background characteristics (e.g., personality and age) may be associate with their beliefs and, in turn, with their intentions and behaviors [36]. Compared to Millennials, Gen Zs may have a higher degree of autonomy in using digital-based platforms and products and are exposed to extensive information more easily through diverse technology [34]. As a result, Gen Z gamers might be accustomed to identifying what they need or want autonomously and tend to rely much less on other people’s opinions than Millennial gamers.

However, our results did not support the significant mediation role of PBC on the relationship between VSPM and purchase intention. We found that while gamers’ belief about their ability to purchase NFPs was positively associated with purchase intention, purchase intention was not significantly associated with gamers’ desire to present themselves in a game. This finding is interesting because PBC, similar to attitude and SN, is considered to be a belief-based measure according to the TPB [11]. Yet, unlike the other two constructs, it was not significantly associated with gamers’ desire for self-presentation in the game. This also contradicts a previous study showing that both intrinsic (e.g., satisfaction) and extrinsic motivations (e.g., prizes or rewards) had strong association with gamers’ PBC when purchasing NFPs [48]. A possible explanation for this finding is that, according to the study, an individual’s perceptions of resources and obstacles can be both internal (e.g., their own abilities) and external (e.g., environmental circumstances) [49]. It is, therefore, likely that although gamers’ high VSPM might convince them to believe that they possess their own abilities or resources (e.g., money) to purchase NFPs, there might be other environmental circumstances (e.g., the availability of their preferred NFPs) of which they are not aware.

Informed by TPB, we also tested the direct association between purchase intention and PBC regarding gamers’ consumption of NFPs. Our results revealed that gamers with a higher intention to purchase NFPs reported a higher consumption rate, a phenomenon supported by consistent findings in various research fields. For example, in the video gaming context, researchers found that gamers’ consumption intention reflects their consumption of playing [27] and in-game purchases [13]. This finding is salient because, as pointed out in previous studies, the relationship between intention and behavior has been largely ignored in existing literature. Our study filled this research gap by revealing the relationship between intention and behavior with empirical evidence.

However, contrary to the tenets of the TPB, the association between PBC and consumption was nonsignificant. Existing research has questioned the predictability of PBC in directly influencing behavior [50]. Instead, PBC has been found to exhibit greater predictive power as a moderator in the intention–behavior relationship, which might explain its nonsignificant direct association with gamers’ purchases [51]. This suggests that how much consumers believe they are able to purchase NFPs may not directly relate to actual purchasing behavior in this context but instead plays a more indirect or conditional role. One possible explanation is that actual purchase behavior is influenced by situational constraints and decision processes that are more directly captured by behavioral intention. This insight is valuable in clarifying the role of PBC within the TPB framework and suggesting that its influence may be context-dependent rather than universally direct when applying TPB in similar contexts.

It is worth noting that there was also a significant difference in the relationship between purchase intention and purchase across Gen Zs and Millennial gamers. While both groups showed a significant positive relationship between purchase intention and consumption, a stronger association was found in the Gen Z group than in the Millennial group. A possible explanation is that Gen Zs, having grown up during a period when the digital gaming industry was more fully developed, are more immersed in video games and more familiar with in-game consumption practices than most Millennials [33]. Thus, Gen Zs might feel more comfortable or familiar with purchasing NFPs they intend to consume compared to Millennials. Indeed, reports suggest that the Gen Z group is more prominent and monetizable gamers than other older generations in the gaming industry [31]. Connecting to the findings of generation-based differences in motivation-cognition, the current model provides a better explanation of gamers’ consumption of NFPs by further specifying the motivation-cognition-intention-behaviors relations based on generation, identifying a boundary condition in which Gen Zs and Millennials are different in motivation, cognition, and behavior pertinent to NFPs consumption.

### Theoretical implications

This study offers several theoretical implications for understanding NFPs consumption. First, the findings contribute to the literature by integrating motivational and cognitive perspectives to explain the formation of consumption behavior. While prior research has often examined either motivational drivers or cognitive evaluations in isolation [1,13,27], the present study demonstrates that motivation is associated with behavior primarily through its influence on cognitive evaluations. This highlights the importance of considering both motivational and cognitive processes when examining decision-making in virtual consumption contexts.

Second, the study provides support for the role of motivated reasoning in shaping belief-based evaluations. Specifically, VSPM is associated with attitude and SN, but not with PBC. This suggests that motivation is more strongly linked to evaluative and social beliefs than to perceptions of control, thereby specifying the mechanism through which motivation relates to decision-making processes. Third, the findings reinforce the structure of TPB by showing that attitude, SN, and PBC are key predictors of purchase intention, while also demonstrating that their formation may be associated with underlying motivational factors. This contributes to the literature by providing a more thorough explanation of the motivation–cognition–intention–behavior process in the context of NFPs consumption.

Finally, the study identifies a boundary condition by highlighting the importance of generational differences as a contextual factor influencing the relationship between motivation and decision-making processes in relation to NFPs consumption behaviors. The results show that Generation Z and Millennials rely on different cognitive pathways, with Generation Z placing greater emphasis on attitude and Millennials being more influenced by SN. This finding suggests that generational cohort may shape how motivation is associated with cognitive evaluations and subsequent behavior, offering additional insight into variations in consumer decision-making within digital environments.

### Practical implications

Our study offers actionable marketing insights into product management within the video game market, focusing on consumer segmentation and key psychological factors that drive NFPs consumption behaviors across diverse esports gamer demographics. Understanding these generational differences is crucial for video game brands and product managers seeking to develop effective branding and marketing strategies for NFPs consumption. Our findings showed that, overall, Gen Zs tend to focus on how they feel and think about the consumption of NFPs; the social influence of others’ opinions seems less important to Gen Z gamers than Millennials.

These generational differences suggest that game developers and marketers should tailor branding and promotional efforts to resonate with distinct consumer segments. For Millennials, whose purchasing behavior is significantly associated with social influence of others’ opinions, game companies should implement strategies that enhance social visibility and peer influence, such as incorporating user-generated content, social proof features (e.g., visible item ownership, popularity indicators), and peer recommendations directly within the game environment. Encouraging community engagement, influencer endorsements, and peer reviews could reinforce positive brand associations and drive NFPs adoption among Millennial gamers.

For Gen Z, who rely more on intrinsic attitudes and personal identity expression, game marketers should emphasize the unique design, creative concepts, and symbolic value of NFPs. Rather than focusing on external validation, video game brands should develop customizable and visually distinctive NFPs that allow players to express individuality, as well as highlight personalization options, aesthetic appeal, and emotional storytelling in their marketing narratives. This approach aligns with broader trends in brand characteristic and identity-driven consumption, making NFPs more appealing to Gen Z consumers who value individuality in their digital self-presentation.

Moreover, although the association between purchase intention and purchase was significant for both generations, a stronger relationship between intention and behavior was observed in Gen Z gamers. This is a valuable insight not only for practitioners of the video game brands but also for those in other virtual marketplaces, as it may indicate that Gen Zs are more willing to take action on their intent to consume compared to Millennials. Given the importance of gamers’ attitudes and SN in relation to purchase intentions, video game brands should design game environments and features that support both self-expression and social interaction. To achieve this goal, brands should facilitate a more socially friendly gaming environment to encourage gamers’ interactivity in games. According to the study, social interaction is essential to influencing people’s desire for virtual self-presentation [24]. Developers could therefore initiate more opportunities for gamers to increase social interaction through their in-game characters. Organizing in-game networking events, for example, may provide gamers’ more opportunities to use their in-game characters or avatars in addition to playing the game.

## Conclusion

The present study sheds light on the motivation-cognition-intention-behavior process regarding NFPs consumption in video game though the theoretical integration of the MRT [12] and the TPB [11]. Our results showed a generational difference in the motivation-cognition-intention-behavior pathways. Our findings suggest that tailored marketing strategies can enhance NFPs consumption across generational segments. Specifically, understanding that Generation Z’s purchase behavior is more self-directed—driven by personal attitudes toward in-game purchases—marketers can focus on personalized, attitude-aligned messaging that resonates with their sense of identity and self-expression in the game. Conversely, Millennials’ purchasing decisions are influenced more by social factors, such as perceived approval or social trends, suggesting that marketing strategies for this group should emphasize community and social validation, perhaps through influencer partnerships or in-game social proof.

### Limitations and future suggestions

This study is not without its limitations. The generalization of this study may be limited, as the composition of the study participants in this biased toward male participants. Given that numerous females enjoy playing online games, results with stronger validity can be presented if the proportion of male and female participants is similar or balanced. Additionally, although our study’s primary purpose was to analyze the purchasing behavior toward NFPs in the context of video game overall, gamers’ motives (e.g., hedonic motivation, social influence, and price value) may differ depending on the type of game and the type of products even among NFPs. Based on the conceptual framework presented in our study, a follow-up study should consider distinguishing between the various game types and products to provide further insight.

Finally, although our model and hypotheses were theoretically driven by TPB and MRT, the cross-sectional SEM approach used here does not permit definitive causal inference among competing causal orderings. Alternative temporal or reciprocal models remain statistically plausible given cross-sectional data. However, what the results do show, in a weaker yet informative sense, is that virtual self-presentation motivation, cognitive evaluations, and purchase outcomes are systematically interrelated and that these patterns of associations differ by generation. These association patterns reveal which paths are stronger across cohorts, providing information that is useful for theory refinement and for generating targeted hypotheses to be tested in longitudinal or experimental designs.

## Supporting information

S1 Appendix Wording of Scale Items. *Note.* VSPM = virtual self-presentation motivation; ATT = attitude; SN = subjective norm; PBC = perceived behavioral control; INT = intention to purchase; ACT = actual purchase. Items used a seven-point format. VSPM, SN, PBC, and INT used Likert-type ranging from strongly disagree to strongly agree.
(DOCX)
