# Supplementary material for: Esports in-game consumption across generations: Integrating motivated reasoning theory and the theory of planned behavior
Source: PLoS One. 2026 May 4;21(5):e0348355. doi: 10.1371/journal.pone.0348355 (PMC13138612; doi:10.1371/journal.pone.0348355)
Supplement: S1 Appendix — Note. VSPM = virtual self-presentation motivation; ATT = attitude; SN = subjective norm; PBC = perceived behavioral control; INT = intention to purchase; ACT = actual purchase. Items used a seven-point format. VSPM, SN, PBC, and INT used Likert-type ranging from strongly disagree to strongly agree. (DOCX) [file pone.0348355.s001.docx]

| Construct | Items |
| --- | --- |
| VSPM | VSPM 1: I want to establish a preferred image for myself in the game I play. |
|  | VSPM 2: I want to present my image in the game I play. |
|  | VSPM 3: I want to project an image of myself in the game I play. |
|  | VSPM 4: I want to give a preferred impression about myself to others in the game I play. |
| ATT | ATT 1: For me, purchasing in-game skin would be: (1=Unpleasant; 7= Pleasant) |
|  | ATT 2: For me, purchasing in-game skin would be: (1=Dull; 7=Entertaining) |
|  | ATT 3: For me, purchasing in-game skin would be: (1=Worthless; 7= Valuable) |
|  | SN 1: Most of the people that are important to me would approve of my purchase of in-game cosmetics or skins. |
| SN | SN 2: Purchasing in-game cosmetics or skins with people close to me (e.g., friends/family) would be something I would like to do. |
|  | SN 3: People close to me (e.g., friends/family) are likely to purchase in-game cosmetics or skins. |
|  | PBC 1: I have complete control of purchasing in-game cosmetics or skins. |
| PBC | PBC 2: For me, buying in-game cosmetics or skins is easy. |
|  | PBC 3: If I want to, I could easily buy in-game cosmetics or skins |
| INT | INT 1: I plan to purchase in-game cosmetics or skins in the future. |
|  | INT 2: I intend to purchase in-game cosmetics or skins in the future. |
|  | INT 3: I expect to purchase in-game cosmetics or skins in the future. |
| ACT | ACT 1: In the last year, how often did you buy in-game cosmetics or skins whenever you played the game? (1=Rarely; 7=Always) |
|  | ACT 2: Currently, how often do you buy in-game cosmetics or skins whenever you play the game? (1=Rarely; 7=Always) |
